# Global partnerships in rare disease research

**DOI:** 10.1242/dmm.052401

**Published:** 2025-07-28

**Authors:** Sanja Rogic, Guillaume Poirier-Morency, Philip Hieter, Paul Pavlidis

**Affiliations:** ^1^ Michael Smith Laboratories, University of British Columbia, 2185 East Mall, Vancouver, BC V6T 1Z4, Canada; ^2^ Department of Psychiatry, University of British Columbia, 2255 Wesbrook Mall, Vancouver, BC V6T 2A1, Canada

**Keywords:** Rare genetic diseases, Model organisms, Functional insights, Gene discovery, International collaboration

## Abstract

Rare diseases collectively impact hundreds of millions worldwide, yet the genetic causes of many remain unknown or poorly understood. Model organisms (MOs) – such as yeast, fly, zebrafish and mouse – provide powerful experimental systems for functional validation of candidate genes and variants, elucidation of gene function and disease mechanisms, and identification of potential therapeutic targets and treatments. However, gaps persist between clinical gene discovery and MO-based research. The Canadian Rare Diseases: Models and Mechanisms (RDMM) Network was established in 2014 to address this gap by linking clinicians with MO researchers through a scientist registry and peer-reviewed funding process. Over the past decade, the RDMM Network has funded over 160 collaborative projects, enabled insights into numerous rare conditions, and led to sustained partnerships and external funding. The RDMM Registry software has been adopted internationally, forming a network of interoperable registries that enable cross-border collaborations and expand access to MO expertise worldwide. Going forward, the Canadian RDMM Network remains committed to sharing its tools, processes and experience to help establish new RDMM-like networks worldwide and invites the global research community to join efforts to accelerate rare disease research.

## Teaser

**Summary:** This article discusses our experience with the Canadian Rare Diseases: Models and Mechanisms (RDMM) Network and the emerging RDMM International network, which aim to accelerate functional research on rare disease genes and promote global collaborations.

## Introduction

It is estimated that over 400 million people globally are affected by a rare disease during their lifetime. Although estimates of the total number of distinct rare diseases vary, most suggest that there are between 6000 and 10,000, with 70-80% having a genetic origin (Ferreira, 2019; Haendel et al., 2020; Kernohan and Boycott, 2024; Nguengang Wakap et al., 2020). Although the past 15 years have seen incredible strides in research into rare diseases, the underlying genetic causes of thousands of these diseases remain unsolved (Bamshad et al., 2019).

Rare disease research has a unique set of challenges. The low prevalence of these conditions makes it difficult to recruit enough patients to establish reliable genotype–phenotype correlations, so often genetic findings must be further validated. Furthermore, rare disease research typically receives less funding than research on more common conditions, as rare diseases affect fewer people and may not attract significant public or private investment. Knowledge and expertise in this field are often limited, fragmented and geographically dispersed. As a result, patients frequently experience prolonged diagnostic journeys, with many remaining undiagnosed even after years of seeking answers (Kernohan and Boycott, 2024).

The first step in establishing a genetic diagnosis for an unknown rare disease is typically performing a DNA-sequencing test. However, even if the test yields candidate causative variants, their pathogenicity must still be validated. In addition, to make meaningful progress towards understanding of the mechanism of the disease and how to treat it, it is crucial to understand the normal biological function of the mutated gene and how the identified variants disrupt it. This is where model organisms (MOs) – such as yeast, worm, fly, zebrafish and mouse – become invaluable. These organisms offer several advantages that make them ideal for advancing rare disease research: they are genetically tractable and easily manipulated, supported by extensive established resources, cost-effective and, most importantly, share evolutionary conserved genes and pathways with humans (Wangler et al., 2017; Yamamoto et al., 2024). However, a disconnect often exists between clinician scientists discovering rare disease-causing genes and MO researchers, who are frequently unaware of these discoveries and the valuable insights their expertise and model systems could offer.

## The Canadian RDMM network

**Fig. 1.**
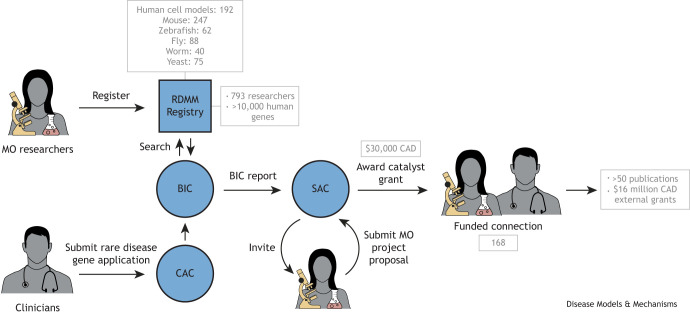
**The Canadian Rare Diseases: Models and Mechanisms (RDMM) Network pathway map.** There are two main entry points into the network. Model organism (MO) researchers join by registering their expertise in the RDMM Registry, whereas clinicians submit a two-page Connection Application that specifies a human gene of interest and outlines the genetic evidence suggesting that the gene (or variants within it) is disease causing. These applications are reviewed by the Clinical Advisory Committee (CAC), which evaluates them using established criteria. Approved applications are forwarded to the Bioinformatics Core (BIC), which uses the Registry to identify suitable MO researchers who can study the proposed gene. The BIC prepares a report for the Scientific Advisory Committee (SAC), which evaluates the identified matches and invites one or more MO scientists to apply for funding. Following the review of the two-page MO Proposal Applications, the SAC awards the successful applicants $30,000 CAD to initiate immediate collaborative experiments.

To bridge this gap, the Canadian Rare Diseases Models and Mechanisms (RDMM) Network was established in 2014, with the goal of connecting rare disease clinicians with Canadian MO scientists able to study equivalent genes and pathways in MOs (Boycott et al., 2020). The Network, built around a registry of MO researchers, uses a committee process to identify and evaluate clinician–MO scientist collaborations and subsequently award $30,000 CAD in catalyst funding. The way the network operates is depicted in Fig. 1. Despite involving three rounds of rigorous scientific review, this process is remarkably efficient, typically completing the journey from initial clinician application to fund disbursement within just 4 weeks.

Over the past decade, the RDMM Network has funded 125 collaborative projects between clinician scientists and MO researchers. Additionally, the network partnered with several foundations to support research projects focused on specific rare diseases – such as Dravet, Tatton-Brown–Rahman, Roifman and Mowat–Wilson syndromes – resulting in 43 additional funded projects and bringing the total to 168. Many of these partnerships have led to significant insights into the molecular mechanisms of the rare disease and, in some cases, the identification of potential therapeutic strategies. One such example is given in Box 1. There are currently over 50 original scientific publications reporting on these findings. Moreover, some of these partnerships have evolved into long-term collaborations supported by 14 large-scale follow-on external grants, totaling $16 million CAD.

Box 1. An example of an impactful Rare Diseases: Models and Mechanisms (RDMM)-funded project Model organisms – such as yeast, worm, fly, zebrafish, mouse and others – offer powerful experimental tools to confirm the pathogenicity of rare disease-causing gene variants, characterize gene function, and identify potential therapeutic targets and treatment strategies. In a recent example, with funding from the RDMM, Heinemann and colleagues generated a yeast model system for the histidyl-tRNA synthetase 1 (HARS1) disease Charcot-Marie-Tooth syndrome (CMT) by replacing yeast ortholog *HTS1* with human wild-type or mutant *HARS1* (Qiu et al., 2023). Expression of four pathogenic *HARS1* mutants caused severe growth defects in yeast that were shown to be caused by the aggregation of unfolded proteins. The growth defect and protein aggregation phenotype of two of the four mutants tested were shown to be rescued by the addition of histidine, revealing histidine supplementation as a potential treatment for these specific CMT variants. The two other mutants were not rescued by histidine, but, in contrast, were further growth inhibited by the addition of histidine. Histidine is currently in a clinical trial to treat patients with Usher syndrome type 3B, caused by a specific *HARS1* variant. Therefore, the yeast model of CMT variants will be useful in considering histidine as a treatment for patients with CMT caused by variants in *HARS1*. Mutated HARS1 can cause widespread errors in translation owing to misincorporation of the wrong amino acid during translation, or through reduced HARS1 activity, which can impair protein synthesis. The yeast model provides a powerful experimental system to further characterize the molecular mechanisms of HARS1 variant toxicity and rescue by amino acid supplementation.

Although the RDMM Registry currently directly supports ten MOs – human cell models (primary patient-derived cells, cell lines derived from induced pluripotent stem cells, and organoids), mouse (*Mus musculus*), rat (*Rattus norvegicus*), zebrafish (*Danio rerio*), frog (*Xenopus tropicalis*), fruit fly (*Drosophila melanogaster*), worm (*Caenorhabditis elegans*), budding yeast (*Saccharomyces cerevisiae*), fission yeast (*Schizosaccharomyces pombe*) and *Escherichia coli* – the Canadian Network accepts research proposals using any MO. The Registry's frequently asked question (FAQ) section advises researchers how to describe their expertise in such cases. When the Scientific Advisory Committee (SAC) evaluates matches found in the Registry, there is no inherent preference for one MO over another; any organism is considered as long as it is scientifically appropriate. If multiple valid proposals involving different organisms are received, the SAC may decide to fund more than one. Among the funded projects, roughly one-third use mouse models, one-third use zebrafish, and the remainder involves other MOs.

## The RDMM Registry

The RDMM Registry serves as the central resource of the RDMM Network (Fig. 1). It is a web-based software platform designed to collect and organize information on MO researchers, enabling comprehensive searches performed by the Bioinformatics Core (BIC) to identify suitable collaborators for applying clinicians.

**Fig. 2.**
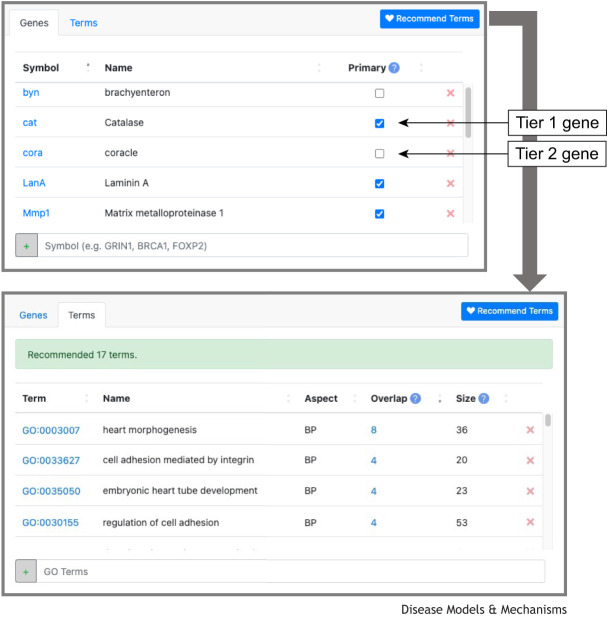
**Gene entry workflow in the RDMM Registry.** (Top) The ‘Genes’ tab displays five of 12 *Drosophila melanogaster* genes in the example user's profile. Genes can be added individually or as a list using the text box at the bottom. Tier 1 genes are marked with a checkmark in the ‘Primary’ column; all others are considered Tier 2. After entering Tier 1 and Tier 2 genes, users can click the ‘Recommended Terms’ button to receive a list of Gene Ontology (GO) term suggestions based on gene overlap. (Bottom) In the ‘Terms’ tab, the ‘Overlap’ column shows how many of the user's Tier 1 and 2 genes are annotated with each suggested GO term, while the ‘Size’ column indicates the total number of genes associated with that term. ‘Aspect’ refers to the category that the GO term belongs to when describing gene function; here, ‘BP’ refers to ‘Biological Process’. Genes linked to accepted terms are added to the user's profile as Tier 3 genes. Users may remove recommended terms or add new ones manually using the text box at the bottom.

The Registry collects several types of information. The Profile page includes a researcher's contact details along with a general overview of their research, including research interests, key publications, and the organ systems, biological pathways and human diseases relevant to their work. Additionally, their specific expertise relevant to the RDMM Network is described by listing genes of interest for each MO they use; this information is of key importance for identifying suitable matches. The listed genes are initially organized into two tiers: Tier 1 represents genes that the researcher is already actively studying; Tier 2 represents genes that the researcher could rapidly set up to study (e.g. paralogs of Tier 1 genes, or members of the same complex or pathway). To expand the gene search space and enhance the potential for matches, the system automatically generates sets of related Tier 3 genes based on Gene Ontology terms (Ashburner et al., 2000; The Gene Ontology Consortium et al., 2023) associated with their Tier 1 and Tier 2 genes. An example of this is given in Fig. 2. MO investigators also have the option to directly choose GO term categories that reflect their expertise as Tier 3 genes. The Registry's real-time statistics are available on the Network's website. As of April 2025, a total of 793 MO researchers across Canada have registered 17,602 unique genes across all ten supported MOs. Based on the Registry's built-in orthology mapping, this corresponds to coverage of 10,062 human genes.

The primary use case for Registry searches by the BIC is to input a human gene and retrieve a list of MO researchers who have that gene or one of its orthologs included in their profiles, leveraging the built-in orthology mapping. The software also supports searches based on researcher name, profile categories and other keywords. The search results depend on the user-selected privacy levels: private data can only be accessed by the BIC, shared data can be accessed by other registered users, and public data can be browsed on the Registry's public search webpage.

The Registry software is open source, with extensive documentation available. It is built as a Java Web application using Spring Boot and Hibernate, with authentication and authorization managed through Spring Security. The backend database service is powered by MySQL, and MO gene lists, along with implemented ontologies, are regularly updated to ensure accuracy and relevance.

## RDMM International

Following the initial success of the RDMM Network model in Canada, we shared our registry software, management plan and processes to support similar emerging networks worldwide. This aims to establish collaborations to expand the pool of MO scientists available to study rare disease genes, regardless of where they were discovered. These international linkages allow cross-border connections between clinicians and basic researchers when a suitable match is not found within a regional network. The first partnerships were established with Europe, Australia and Japan. These were further expanded to include USA-based ModelMatcher, a global matchmaking platform for facilitating collaborative research on rare and undiagnosed diseases (Harnish et al., 2022) and, more recently, a network in Singapore (Box 2). To date, 11 cross-border clinician–MO scientist connections have been established through the Canadian RDMM Network, involving ten Canadian MO researchers (nine of them receiving Canadian RDMM funding) and one Australian MO researcher (funded by the Australian Functional Genomics Network), with additional connections made between other partner networks. Details on all RDMM International partners – including information about their organizational structures, committee processes, funding models, registry statistics and cross-border collaboration rules – can be found on the RDMM International website.

Box 2. RDMM International partners Several international rare disease networks have adopted the procedures, committee structure and registry software developed by the Canadian RDMM Network (with the exception of Japan's J-RDMM, which created its own version of the software based on the RDMM Registry). Each of these networks has provided funding to support established connections between clinicians and model organism (MO) researchers. These networks are as follows:

- The European Rare Disease Models & Mechanisms Network (RDMM-Europe), which was operational from 2019 to 2022 as part of the European Commission-funded Solve-RD research project (Ellwanger et al., 2024). Although the RDMM-Europe is not funding any more projects, its registry is still functional and searchable.
- Australian Functional Genomics Network (AFGN), as part of the Australian Genomics Health Alliance and funded by the Medical Research Future Fund during the period 2021-2026.
- The Japanese Rare Disease Models & Mechanisms Network (J-RDMM), which was launched in 2017 as part of the Initiative on Rare and Undiagnosed Diseases.
- Singapore Rare Disease Models and Mechanisms (SG-RDMM), which was established in 2022 by the SingHealth Duke-NUS Genomic Medicine Centre.

The Canadian RDMM, RDMM-Europe, AFGN and SG-RDMM registries are interoperable through secure application programming interface (API) connections, enabling cross-network data querying and retrieval.

ModelMatcher (Harnish et al., 2022), based in the USA, represents a different kind of network partner, as it does not use a committee review process or provide funding for established connections. Instead, it is designed as a global matchmaking platform to facilitate collaborative research on rare and undiagnosed diseases. Its Scientist Registry, built on the RDMM Registry software, supports the registration of not only MO researchers but also a broader range of scientists, such as cell biologists, biochemists, structural biologists and bioinformaticians. Anyone seeking to connect with registered scientists (for example, clinicians, patients or funding agencies) can do so using ModelMatcher's Match Dashboard. ModelMatcher is connected to other international network registries through secure API connections, but its access is limited to data with ‘public’ privacy level.
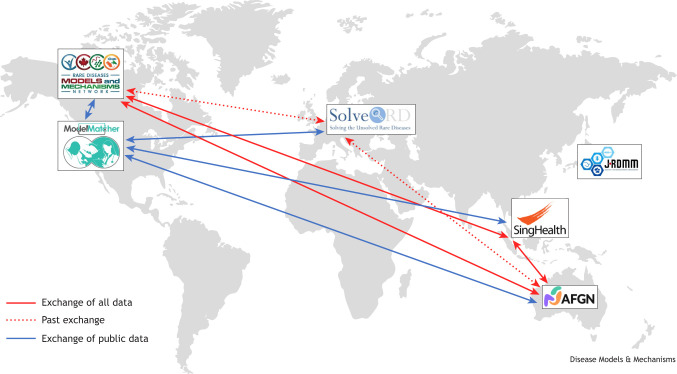

To enable international linkages, the Canadian RDMM Registry software was made portable, customizable and linkable to other installations of the platform. Seamless data sharing between different installations is achieved through the platform's application programming interface (API), which enables remote querying and data retrieval while adhering to defined security settings. The API also allows third-party applications and external resources to access the Registry's public data, enabling integration with other research tools.

Furthermore, we made the user interface highly flexible and customizable, enabling each installation to define its own descriptors and categories for user profiles utilizing formal ontologies. Enhanced security and data privacy features make it easy to select global default settings for a specific installation, including international data sharing, while still offering users the flexibility to modify these according to their preferences. Further upgrades were implemented to improve system performance, customization of website documentation and FAQs, and ease of maintenance.

The Registry software's flexible and modular design enables its use beyond the rare disease domain. For instance, the Canadian Pediatric Cancer Models and Mechanisms Network has adapted the software to create a directory of preclinical cancer researchers with linkages to public data in other RDMM International registries.

## Challenges in establishing rare disease partnership networks

When establishing the Canadian RDMM Network, a critical decision was made to invest substantial effort into developing the Registry software, as we recognized the importance of minimizing data entry burden for the MO community while at the same time enabling the BIC to efficiently search for potential connections. Another key organizational task was assembling the Clinical Advisory Committee (CAC) and SAC – six to ten member committees essential to the efficient functioning of the Network. This required recruiting members committed to participating in bimonthly meetings and conducting rapid, timely scientific reviews, while ensuring balanced representation across MOs and geographic regions. Each regional RDMM network has their own procedures for recruiting CAC and SAC members.

Additionally, a significant challenge lies in successfully recruiting the entire MO research community within a given RDMM network region and encouraging researchers to comprehensively enter their research expertise and genes of interest into the Registry. In Canada, this was accomplished by having provincial MO community leaders and SAC members compile lists of Canadian scientists from their own professional circles and contact them directly. This was followed by several email campaigns reminding the registered MO researchers to complete or update their registry profiles. Similarly, clinicians discovering rare disease genes needed to be informed and encouraged, through their respective clinical networks, to submit rare disease gene Connection Applications to the CAC. These efforts remain ongoing for the Canadian RDMM Network. Continued participation from both MO researchers and clinicians is essential to realize the full potential of the RDMM framework, and we encourage both communities – within and beyond Canada – to connect with their regional nodes or the global ModelMatcher initiative.

Regional RDMM networks have the autonomy to conform with local guidelines – such as legal requirements for establishing collaborations or protecting data – and to accommodate different national funding schemes. This flexibility ensures that these aspects do not impact the operations of other RDMM networks.

## The way forward

Currently in its third funding cycle, the Canadian RDMM Network continues to catalyze collaborations between clinicians and MO researchers in the early stages after discovery of the rare disease gene or variant. The network also increases awareness of the critical role of fundamental research in understanding the biological mechanisms underlying human disease. Despite the RDMM Registry's widespread adoption by Canadian MO scientists with expertise covering roughly half of the human genome and the access to additional expertise through our international partners, some connection applications and their target rare disease genes remain unmatched. Expanding the RDMM International network will increase the likelihood that every rare disease gene that enters the individual networks, regardless of the point of entry, is matched with the scientist best suited to functionally characterize it. A coordinated, large-scale global effort is the best way forward to elucidate the genetic origins of poorly understood rare diseases and ultimately enhance diagnosis, care and treatment options for those affected.

Drawing from a decade's experience, the Canadian RDMM Network remains dedicated to fostering global partnerships and driving the expansion the RDMM International Network. We are committed to assisting with the development of additional RDMM-like networks by sharing our procedures, policies, software and experience. We invite research groups and initiatives around the world to join us in advancing rare disease research and transforming patient care.
